# DIRECT CARE WORKERS EMPLOYED BY PRIVATE HOUSEHOLDS: A DISTINCT LABOR FORCE

**DOI:** 10.1093/geroni/igz038.2582

**Published:** 2019-11-08

**Authors:** Christopher M Kelly, Jerome Deichert, Lyn Holley

**Affiliations:** 1 University of Nebraska at Omaha, Omaha, Nebraska, United States; 2 University of Nebraska, Omaha, United States

## Abstract

Purpose: This study tracks the growing number of direct care workers (DCWs) employed by private households and describes the differences between this often ignored labor force and DCWs employed by agencies. Design and Methods: Data were from the 1% Public Use Microdata Sample (PUMS) of the 2000 and 2017 American Community Survey (ACS). Logistic regression was used to compare demographic and employment characteristics of DCWs employed by private households and DCWs employed by agencies, which include outpatient care centers, home health care services, and individual and family services. Results: Between 2000 and 2017, the number of DCWs employed by private households in the U.S. increased 32% and the majority of this growth was since 2007. Compared to DCWs employed by agencies, DCWs employed by private households were more likely to be over age 65, white, unmarried, have higher educational attainment, be more likely to be in poverty, receive health insurance from Medicare or direct-pay. DCWs employed by private households were less likely to be under age 25, nonwhite, Hispanic, speak a language other than English, work year-round and full-time, receive health insurance from an employer or through Medicaid, and have a disability. Implications: DCWs employed by private households represent a small, but growing proportion of the long-term care (LTC) workforce in the U.S. Further, these workers are distinct within the LTC workforce. This has important implications both for DCWs and for families, particularly those with limited LTC options due to location, financial resources, family support, or other factors.

